# *De novo* transcriptomic data of salt tolerant halophytes *Dichnathium annulatum* (Forssk.) stapf and *Urochondra setulosa* (Trin.) C.E.Hubb.

**DOI:** 10.1016/j.dib.2021.107536

**Published:** 2021-11-01

**Authors:** Anita Mann, Naresh Kumar, Ashwani Kumar, Charu Lata, Arvind Kumar, B.L. Meena, Sonam Gaba, Monendra Grover

**Affiliations:** a ICAR-Central Soil Salinity Research Institute, Karnal, Haryana 132001, India; b ICAR-Indian Agricultural Statistical Research Institute, New Delhi 110011 India

**Keywords:** Transcriptomics, Salinity, Halophytes, DEGs, SSR

## Abstract

Two halophytes, *Dichanthium annulatum* (moderately salt tolerant) and *Urochondra setulosa* (highly salt tolerant) were selected to generate transcriptome at different salinity levels. Sequencing of RNA samples was done on Illumina-Hi-Seq platform for *de novo* transcriptome assembly from the leaf tissues of *D. annulatum* at salinity of ECe ∼30 dS/m and of *U. setulosa* at three salt levels (*i.e.* ECe ∼30, ∼40 and ∼50 dS/m). DESeq was used for identification of differentially expressed transcripts and a total of 267,196 and 384,442 transcripts were assembled through Trinity in both the plants respectively. A total of 32,246 and 25,479 SSRs were identified respectively in both the plants using MISA perl script with mono and tri-nucleotide repeats as most common motif.

## Specifications Table

**Table:** 

Subject	Biological Sciences
Specific subject area	Transcriptomics
Type of data	Table Chart Figure
How data were acquired	Illumina HiSeq
Data format	Raw Sequencing Reads (FastQ)
Parameters for data collection	Total RNA was isolated from leaves of both the halophytes, *D. annulatum* at EC 30dS/m and *U. setulosa* at salinity treatments of EC 30, 40 and 50 dS/m (∼ 300, 400 and 500 mM NaCl) for sequencing.
Description of data collection	Leaves of both the plants were collected in ice and RNA was isolated with one set of control and different salinity treatments in two replications each separately for both the plants. Sequencing was performed on Illumina-HiSeq platform. The RNAseq libraries were prepared with Illumina-compatible NEBNext® Ultra™ Directional RNA Library Prep Kit. Processed reads were assembled using graph-based approach by Trinity program. Clustering of the assembled transcripts based on sequence similarity was performed using CD-HIT-EST. Processed reads were aligned back to the final assembly using Bowtie with end to end parameters. DESeq, was used for differential expression analysis. SSRs were identified using MISA.
Data source location	Division of Crop Improvement, ICAR-Central Soil Salinity Research Institute, Karnal-132001 Haryana, India
Data accessibility	The datasets generated are deposited in the National Center for Biotechnology Information (NCBI) Sequence Read Archive under Bioproject PRJNA561259 for *U. setulosa* and PRJNA665324 for *D. annulatum*. https://www.ncbi.nlm.nih.gov/bioproject/PRJNA561259 https://www.ncbi.nlm.nih.gov/bioproject/PRJNA665324 The sequencing reads of *D. annulatum* control set are available in NCBI SRA accession number SRX9180586 and for salt treatment with accession number SRX9180587 https://www.ncbi.nlm.nih.gov/sra/SRX9180586 https://www.ncbi.nlm.nih.gov/sra/SRX9180587 The sequencing reads of *U. setulosa* control set are available in NCBI SRA accession number SRX6746126 and for salt treatments of EC 30, 40 and 50 dS/m are avaialbe with accession numbers SRX6746125, SRX6746128, SRX6746127 respectively. https://www.ncbi.nlm.nih.gov/sra/?term=SRX6746126 https://www.ncbi.nlm.nih.gov/sra/?term=SRX6746125 https://www.ncbi.nlm.nih.gov/sra/?term=SRX6746128 https://www.ncbi.nlm.nih.gov/sra/?term=SRX6746127 The data of differentially expreseed genes of both *D. annulatum* and *U. setulosa* has been deposited in the repository at Mendeley data and is available at https://data.mendeley.com//datasets/c9zwjncxb4/1
Related research article	Mann, A., Kumar, N., Kumar, A. et al. de novo transcriptomic profiling of differentially expressed genes in grass halophyte *Urochondra setulosa* under high salinity. Sci Rep 11, (2021). 5548 https://doi.org/10.1038/s41598-021-85220-7

## Value of the Data

• These halophytes, *Dichanthium annulatum* and *Urochondra setulosa,* are naturally salt loving plants, where the earlier is moderately salt tolerant surviving upto EC 30 dS/m (∼300 mM NaCl) while the later is highly salt tolerant with salt tolerance upto EC 50 mM (∼500 mM NaCl). There is no reference genome available for these two halophytic plants, hence, the transcriptomic information generated here will be useful for further identification of genes, pathways, mechanism at high salinity in related species.
• The studied halophytes are important dessert plants with economic value as well and having potential in desalinating waste lands. The information generated is valuable for plant researchers working in abiotic stress.
• For crop improvement programmes, this information might be useful in development of markers/QTLs, genic-markers, SNPs or different transcription factors involved in various pathways operating at high salt levels which is need of the time for enhancing crop productivity in changing climatic situations.

## 1 Data Description

**Fig. 1:**
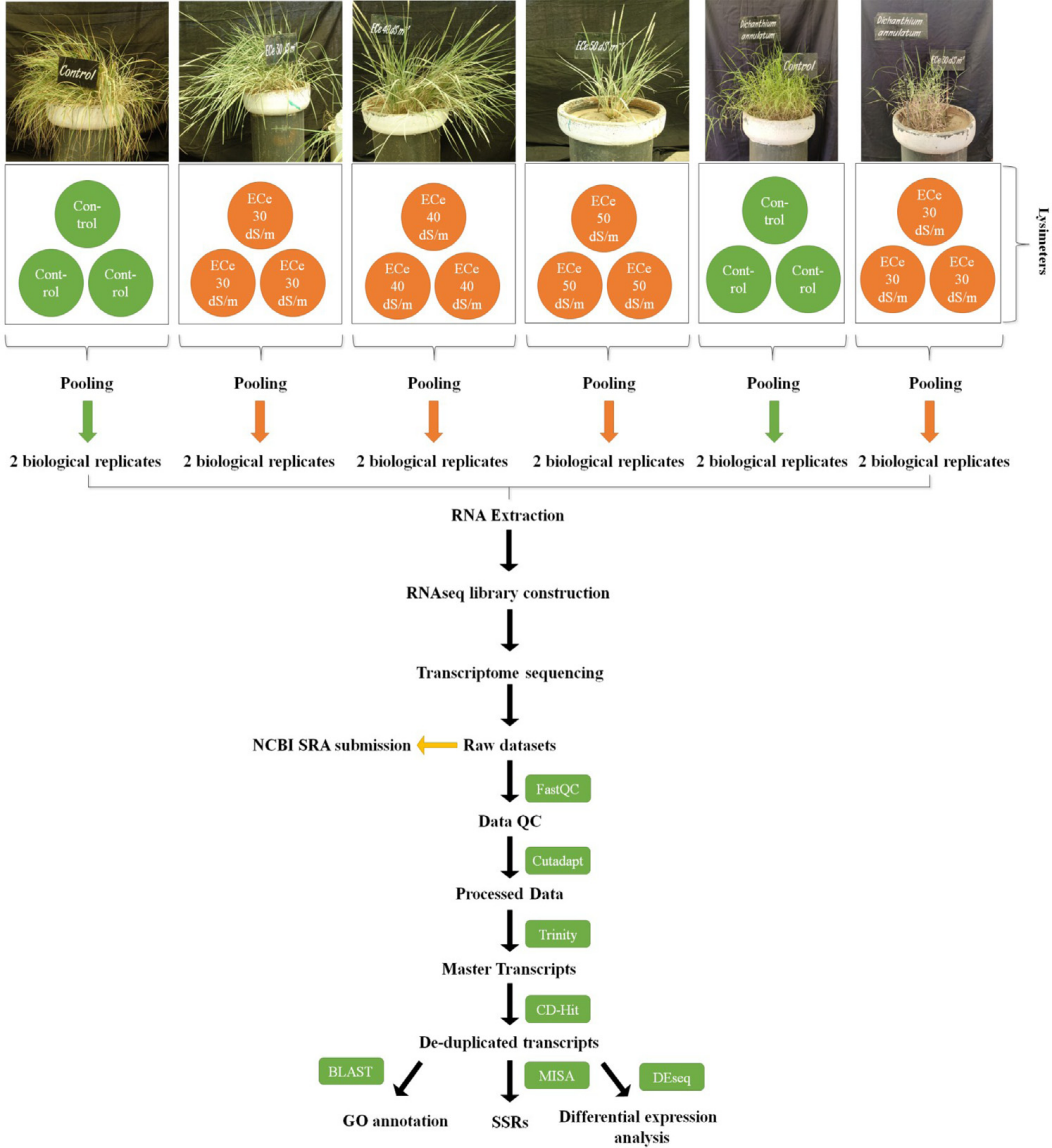
A schematic overview of experimental design and transcriptomic data analysis pipeline in *U. setulosa and D. annulatum*.

**Fig. 2:**
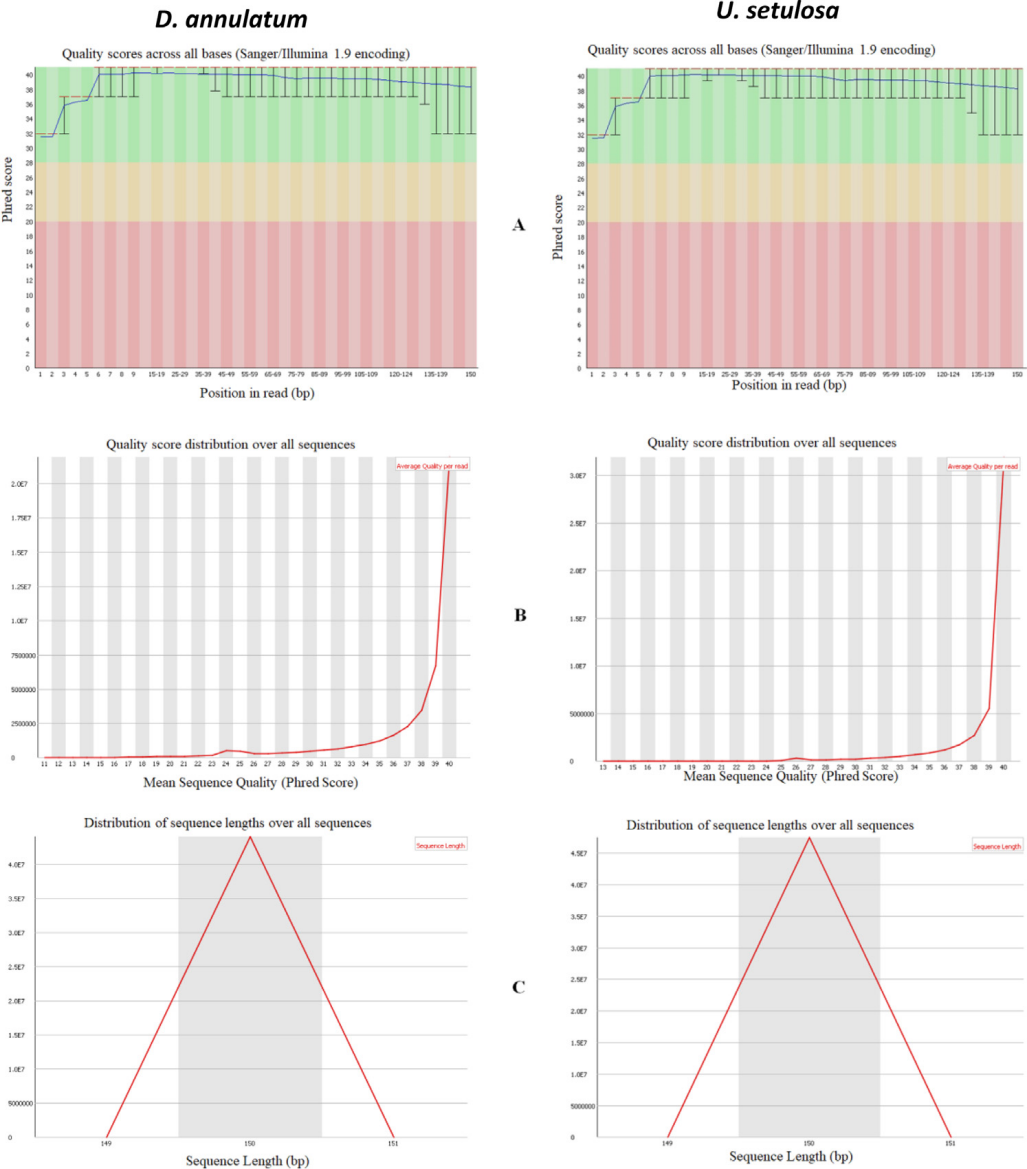
**Visualization of fastQC of *D. annulatum* and *U. setulosa* transcriptomic data. (**A) Per base sequence quality (B) Per sequence quality scores (C) Sequence length distribution.

**Fig. 3:**
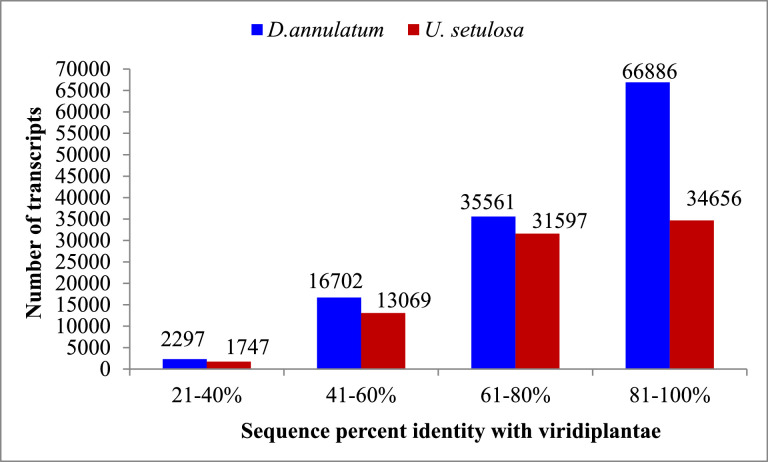
Sequence percent similarity distribution plot in *D. annulatum* and *U. setulosa* aligned against Viridiplantae data

**Fig. 4:**
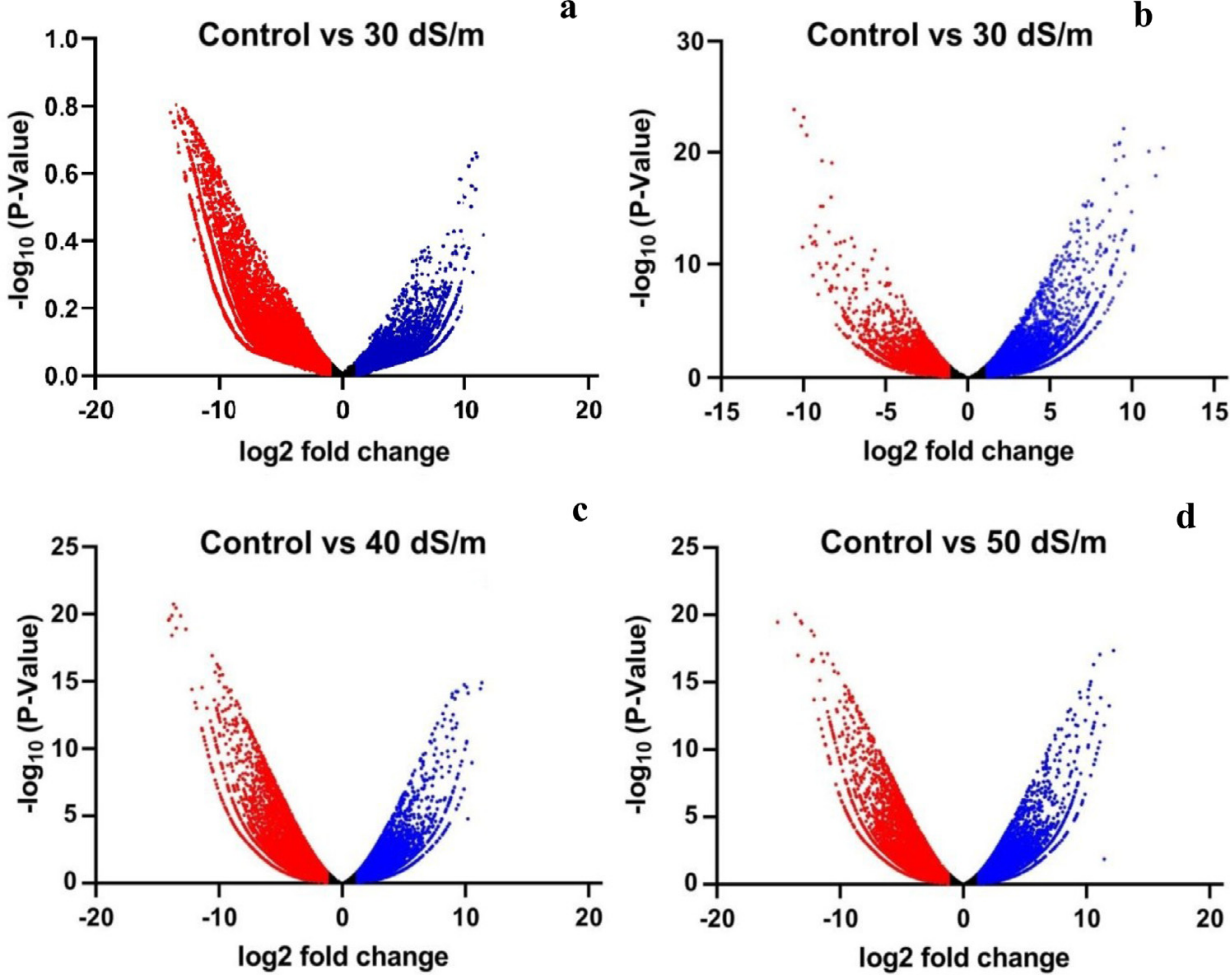
Volcano plot of differentially expressed genes (DEGs) for each salt treatment in *D. annulatum* (a) and *U. setulosa* (b, c, d)**.** Red dots represent down-regulated, blue for up-regulated and black dots represent neutrally-regulated genes in each plot.

**Fig. 5:**
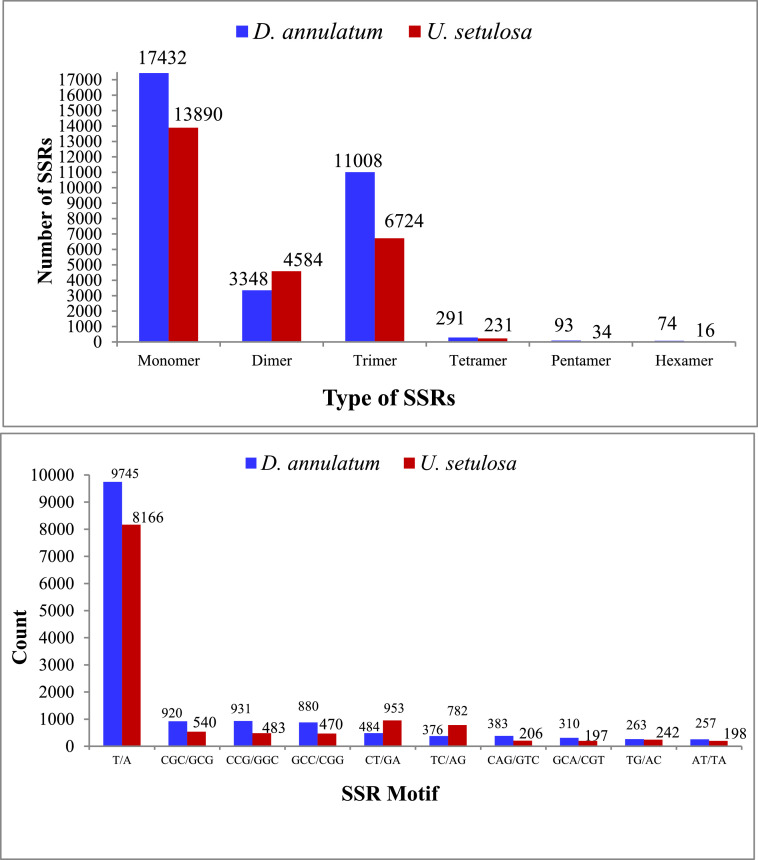
Description and Motif distribution of SSRs in *D. annulatum* and *U. setulosa*.

An aliquot of RNA isolated from leaves of control and salt treated both the halophytic plants was run on Agilent TapeStation to check RNA integrity. All the samples were having RIN (RNA integrity number) values more than 7 (Table 1). A schematic overview of experimental design and transcriptomic data analysis pipeline used in this work has been shown in Fig. 1. After sequencing, a total of 44.3-49.6 million paired end reads were obtained from 8 RNA libraries in *Urochondra* and 4 libraries in *D. annulatum***.** The quality of data was assessed using FastQC (https://www.bioinformatics.babraham.ac.uk/projects/fastqc/) and important parameters such as mean quality scores per position, per sequence quality scores, GC content distribution and read length distribution were measured (Fig. 2). The phred quality score per position of all libraries was higher than 30 with normal distribution of GC content. This analysis showed that there is no sequence contamination during sequencing. After trimming the raw reads using Cutadapt [1], an average of 96% of high quality data was retained and more than 90% of data were found to align with clustered transcripts. The details of the transcriptomic data of *D. annulatum* and *U. setulosa* are shown in Table 2. The trinity assembly of high quality reads resulted in 352.78 million transcripts in *Urochondra* samples, further clustering into 282,719 transcripts with average length of 1,259 bp and N50 value of 1,819 bp whereas in *D. annulatum*, 267.19 million transcripts were clustered into 188,353 transcripts with average length of 864.2 bp and N50 value of 1,100. Trinity [2] combines the overlapping reads of a given length and quality into longer contig sequences without gaps. Main properties of assembled contigs including average length, N50 length, maximum and minimum length were calculated. Contigs shorter than 300 bp were not counted since a characterized protein domain may be either lacking in shorter sequences or we may not find any significant match for such sequences. BLASTing was done against “*viridiplantae*” for functional annotation of clustered transcripts. A total of 65.52% of the transcripts were annotated in *Urochondra* while 64.47% transcripts were annotated in *D. annulatum*. Transcripts with matching e-value less than e-5 and minimum 30% similarity were assigned with a homolog protein from other organisms. The E-value distribution of the transcripts showed that 47.99% of aligned transcripts had similarity with an E-value range of 1e-05 to 1e-60, whereas the remaining 52% of the homologous sequences ranged from 1e-5 to 0. The similarity distribution of transcripts showed that 55.07% and 42.75% of the sequences had a similarity higher than 80% in *D. annulatum* and *U. setulosa* respectively and remaining 44.9 and 57% of the sequences in each plant had a similarity in range of 21-80% (Fig. 3). We also analysed the novel gene expression patterns by performing analyses of the differentially expressed genes (DEGs) related to salinity/salt tolerance. DESeq [3] normalized expression values were used to calculate fold change for a given transcript. The regulation for each transcript was assigned based on log2fold change. Being *de novo*, the transcripts showing log2fold change less than -1 were counted as down regulated and the values more than 1 were represented as up regulated. The data of differentially expressed genes with their expression levels at different salt concentrations in both the plants is available at Mendeley Data (https://data.mendeley.com//datasets/c9zwjncxb4/1). In *Urochondra*, out of total 345,729 transcripts, 68,455 genes were up-regulated and 69,759 were down-regulated. The volcano plots were plotted for each saline treatment as shown in Fig. 4. We identified a total of 8,074 DEGs commonly up-regulated and 1,929 DEGs commonly down-regulated in *U. setulosa* between the three stress treatments (*p* ≤0.05)*.* In addition, 1,065 transcripts (2.8%) were commonly up-regulated at EC 30 and 40 dS/m, 11,209 (29.2%) transcripts between EC 40 and 50 dS/m and 1,627 transcripts (4.2%) at EC 30 and 50 dS/m. Similarly, 1,234 transcripts (2.4%) were down-regulated at salinity levels of EC 30 and 40 dS/m, 18,151 transcripts (35.7%) at EC 40 and 50 dS/m and only 842 transcripts (1.7%) were commonly down-regulated at EC 30 and 50 dS/m. Similarly in *D. annulatum*, 147,851 transcripts were differentially expressed in both control and salt treated samples with 29,482 and 42,425 up and down regulated transcripts. A total of 7,998 genes were differentially expressed (p≤0.05) w.r.t. saline treatment of EC 30 dS/m. The co-expression analysis of differentially expressed genes in both the halophytes can provide an insight into common salt tolerance governing genes. A total of 282,719 *Urochondra* sequences with 353,398,750 bp were mined for SSR prediction. Out of these, 25,479 SSRs were identified in 21,255 sequences with a frequency of 17.18%. Similarly, in *Dichanthium*, a total of 188,353 SSRs were examined with total size of 162,771,602 bp. From these, 32,246 SSRs were identified in 27,430 sequences with a frequency of 14.56%. However 3,542 (16.66%) transcripts in *Urochondra* and 4,114 (14.99%) in *Dichanthium* were found to have more than one SSRs. 1,401 and 1,060 SSRs were identified in compound form in *U. setulosa* and *D. annulatum* respectively while the remaining were the perfect SSRs. The identified SSRs were classified as per the criteria proposed by Weber [4] and it was observed that mononucleotide (17,432 and 13,890) and tri-nucleotide repeats (11,008 and 6,724) were the most abundant motifs in both the plants representing about ∼54% and 34.13%, 26.39% of the total SSRs respectively (Fig. 5) followed by dinucleotide (10.38;17.99%), tetra-nucleotide (0.90%), penta-nucleotide (0.28%, 0.13%) and hexa-nucleotide (0.22, 0.06%) in *D. annulatum* and *U. setulosa* respectively. Motif type prediction revealed T/A as the most abundant motif in both the halophytes followed by CT/GA and TC/AG in *Urochondra* with CCG/GGC and CGC/GCG in *Dichanthium* (Fig. 5).

**Table 1:** RNA concentration and purity of samples estimated using Nanodrop and Qubit fluorometer.

		NanoDrop QC					Qubit QC			Sample quality control
S. No.	Sample ID	ng/µl	260/280	260/230	Volume (µl)	Yield (ng)	Qubit Conc. (ng/µl)	Volume (µl)	Yield (ng)	RIN
1	DC-R1	586.4	2.12	2.31	20	11728	564	20	11280	7.9
2	DC-R2	256.1	2.14	2.16	20	5122	204	20	4080	7.7
3	DT1-R1	158.4	2.14	1.75	20	3168	145.2	20	2904	7.5
4	DT1-R2	224.5	2.15	1.69	20	4490	188	20	3760	7.4
5	UC-R1	601.50	2.15	2.07	20	12030	570.9	20	11418	7.6
6	UC-R2	668.40	2.15	2.34	20	13368	558.8	20	11176	7.3
7	UT1-R1	676.00	2.14	2.07	20	13520	619.3	20	12386	8.1
8	UT1-R2	744.90	2.20	2.19	20	14898	678.0	20	13560	8.5
9	UT2-R1	248.20	2.17	2.05	20	4964	291.0	20	5820	7.8
10	UT2-R2	165.60	2.16	0.97	20	3312	174.3	20	3486	7.6
11	UT3-R1	92.70	2.16	0.57	20	1854	105.4	20	2108	7.6
12	UT3-R2	91.30	2.15	1.46	20	1826	97.8	20	1956	8

D=Dichanthium annulatum; U=Urochondra setulosa; C = Control; T1= EC 30 dS/m; T2= EC 40 dS/m; T3=EC 50 dS/m.

**Table 2:** Details of the transcriptomic data of *D. annulatum* and *U. setulosa*.

Sample ID	Number of RAW reads	Read length (bp)	GC %	Number of clean reads	Percentage of clean reads	Accession number (Biosample)	SRA accession number
DC-R1	38783223	150	54	37744692	97.32	SAMN16250549	SRX9180586
DC-R2	44940563	150	54	43699837	97.24	SAMN16250549	SRX9180586
DT1-R1	43816048	150	52	41878827	95.58	SAMN16250550	SRX9180587
DT1-R2	43999995	150	52	42344295	96.24	SAMN16250550	SRX9180587
UC-R1	47194657	150	53	45713802	96.86	SAMN12612767	SRX6746126
UC-R2	49603777	150	52	47909997	96.59	SAMN12612767	SRX6746126
UT1-R1	44329148	150	51	42730836	96.39	SAMN12612768	SRX6746125
UT1-R2	47434961	150	52	46005424	96.99	SAMN12612768	SRX6746125
UT2-R1	44331551	150	52	41927002	94.58	SAMN12612769	SRX6746128
UT2-R2	45720932	150	52	43329018	94.77	SAMN12612769	SRX6746128
UT3-R1	45649715	150	51	42978094	94.15	SAMN12612770	SRX6746127
UT3-R2	44442832	150	51	42186543	94.92	SAMN12612770	SRX6746127

D=Dichanthium annulatum; U=Urochondra setulosa; C = Control; T1= EC 30 dS/m; T2= EC 40 dS/m; T3=EC 50 dS/m.

## 2 Experimental Design, Materials and Methods

Root slips of both the halophytes *Dichanthium annulatum* and *Urochondra setulosa* were established in lysimeters (40 × 70 cm) filled with sandy soil under open net house at ICAR - Central Soil Salinity Research Institute (CSSRI), Karnal, Haryana, India (29°43`N, 76°58`E;245 m above the mean sea level). Chloride dominated [3(Cl):1(SO_4_)] saline irrigation water was applied after 30 days of establishment of plants and continued till desired level of ECe 30, 40 & 50 dS/m. These saline levels were maintained regularly and after six months, final saline treatments were applied at flowering stage and leaves were harvested after 48 hours for RNA isolation. Three replicates were pooled to make one biological replicate and two biological replicates (pooled) per treatment were used for RNA library construction and further transcriptome profiling in both the plant types.

### 2.1 RNAseq library preparation and RNA sequencing

Total RNA was isolated using Qiagen RNeasy plant mini kit which was quantified on Nanodrop Spectrophotometer while RNA purity was checked on Nanodrop Spectrophotometer. Illumina-compatible NEBNext® Ultra™ Directional RNA Library Prep Kit (NEB, USA) was used for RNAseq libraries as per manufacturer's instructions. cDNA-library was prepared following standard Illumina protocol with synthesis of first strand using Actinomycin D (Gibco, life technologies, CA, USA) followed by second strand synthesis. Double-stranded cDNA was purified using HighPrep magnetic beads (Magbio Genomics Inc, USA) and after end-repairing and adenylation, it was ligated to Illumina multiplex barcode adapters as per NEBNext® Ultra™ Directional RNA Library Prep Kit protocol.

Indexing-PCR of adapter-ligated cDNA was followed for enrichment of adapter-ligated fragments. The reaction was carried out at (37˚C for 15 mins, with denaturation at 98˚C for 30 sec followed by 15 cycles of 98˚C for 10 sec, 65˚C for 75 sec and 65˚C for 5 min. The sequence library (final PCR product), thus constructed was purified with HighPrep beadswith quality check on Qubit fluorometer (Thermo Fisher Scientific, MA, USA) and fragment size distribution was analysed on Agilent 2200 Tapestation.

The constructed RNAseq libraries were used for sequencing on Illumina HiSeq sequencer at Genotypic Technology, Bangalore (India) to generate 150 base pair length paired-end reads. On an average 460.88 and 428.85 million raw sequencing reads were generated in *U. setulosa* and *D. annulatum* respectively which were processed for quality assessment and low-quality filtering before the assembly. The raw data generated was checked for the quality using FastQC (https://www.bioinformatics.babraham.ac.uk/projects/fastqc/). The reads were processed for quality assessment and low quality filtering before the assembly. Pre-processing of the data was done with Cutadapt which includes removing the adapter sequences and low quality bases (<q30). Graph-based approach was used for assembling of processed reads through Trinity program [2] by combining the overlapping reads of a given length and quality into longer contig sequences without gaps.. Based on sequence similarity, assembled transcripts were clustered using CD-HIT-EST [5] with 95% similarity between the sequences which reduces the redundancy without exclusion of sequence diversity. These clustered transcripts were used further for annotation and differential expression analysis. To evaluate the read content and assess the quality of the assembly, Bowtie [6] was used for final assembly through back alignment of processed reads with end to end parameters. Differential expression of transcripts was analysed using DESeq [3]. Sequencing (uneven library size/depth) bias among the samples was removed by library normalization using size factor calculation in DESeq.

### 2.2 SSR marker detection

MISA (MicroSatellite identification tool) perl script was used for mining Simple Sequence Repeats (SSR) in each transcript sequence. Sequence repeats with length and motif type were identified with recommended default protocol of MISA [7].

The commands used for all these programs are available in Supplementary table 1.

## Ethics Statement

All the authors hereby declare that all the experiments were conducted while maintaining all ethical rules and regulations. None of the studies included humans or animals.

## CRediT authorship contribution statement

**Anita Mann:** Conceptualization, Project administration, Writing – original draft. **Naresh Kumar:** Formal analysis, Writing – original draft. **Ashwani Kumar:** Data curation. **Charu Lata:** Formal analysis. **Arvind Kumar:** Visualization. **B.L. Meena:** Methodology. **Sonam Gaba:** Data curation. **Monendra Grover:** Data curation.

## Declaration of Competing Interest

The authors declare no competing financial interests which could influence the work reported in this article.

## Appendix Supplementary materials

Image, application 1
