# Supplementary material for: De novo transcriptomic data of salt tolerant halophytes Dichnathium annulatum (Forssk.) stapf and Urochondra setulosa (Trin.) C.E.Hubb
Source: Data Brief. 2021 Nov 1;39:107536. doi: 10.1016/j.dib.2021.107536 (PMC8581266; doi:10.1016/j.dib.2021.107536)
Supplement: Supplementary file 1 [file mmc1.docx]

### Supplementary table 1: List of command lines for various programs used for transcriptomic data generation

| **FastQC v0.11.3** | *fastqc Input.fastq.gz -t 30 -o fastqc_reports/* |
| --- | --- |
| **Cutadapt-v1.8.3** | *cutadapt -a AGATCGGAAGAGC -A GCTCTTCCGATCT -o Out_R1.fastq.gz -p Out_R2.fastq.gz-m 20 -q 30 Input_R1.fastq.gz Input_R2_.fastq.gz,* |
| **Trinityrnaseq-v2.2.0** | *Trinity --seqType fq --SS_lib_type RF --full_cleanup --max_memory 500G --left Input_R1.fastq.gz --right Input_R2.fastq.gz --output Assembled_Transcript --CPU 15* |
| **CD-HIT version 4.6** | *cd-hit-est -i Assembled_Transcript.fasta -o Assembled_Transcript_CD-HIT.fasta -aS 0.90 -T 25* |
| **Bowtie2-2.2.5** | *bowtie2 --end-to-end -p 5 -x Assembled_Transcripts_CD-Hit -1 in_forward.fastq.gz -2in_reverse.fastq.gz -S out.sam* |
| **MISA perl script** | perl [misa.pl](http://misa.pl/) Assembly.fasta |
